# Prognostic Significance of Homocysteine Level on Neurological Outcome in Brain Arteriovenous Malformations

**DOI:** 10.1155/2020/6661475

**Published:** 2020-11-30

**Authors:** Fa Lin, Chaofan Zeng, Peicong Ge, Dong Zhang, Shuo Wang, Jizong Zhao

**Affiliations:** ^1^Department of Neurosurgery, Beijing Tiantan Hospital, Capital Medical University, No. 119 South Fourth Ring Rd West, Fengtai District, Beijing 100070, China; ^2^China National Clinical Research Center for Neurological Diseases, Beijing 100070, China; ^3^Center of Stroke, Beijing Institute for Brain Disorders, Beijing 100070, China; ^4^Beijing Key Laboratory of Translational Medicine for Cerebrovascular Disease, Beijing 100070, China; ^5^Savaid Medical School, University of the Chinese Academy of Sciences, Beijing, China

## Abstract

**Objective:**

We aimed to investigate the serum homocysteine (Hcy) level in patients with brain arteriovenous malformation (bAVM) and their impact on neurological outcome during hospitalization.

**Method:**

We retrospectively reviewed patients diagnosed with bAVMs in Beijing Tiantan Hospital from January 2019 to August 2020. Patients were divided into two groups according to the mRS (modified Rankin Scale) score at discharge. Clinical and laboratory characteristics were compared. Logistic regression analyses were performed to identify the potential risk factors for short-term neurological outcome.

**Results:**

A total of 175 bAVM patients were enrolled in the study, including 139 patients with favorable outcome (mRS ≤ 2) and 36 patients with unfavorable outcome (mRS > 2). Hyperhomocysteinemia was identified in 32.6% of cases (*n* = 57). Serum Hcy level was related to seizure manifestation (*P* = 0.034) and short-term neurological outcome (*P* = 0.027). Logistic regression analysis showed that serum glucose (OR 1.897, 95% CI 1.115-3.229; *P* = 0.018) and Hcy level (OR 0.838, 95% CI 0.720-0.976; *P* = 0.023) were significantly associated with short-term disability.

**Conclusion:**

Our results indicated that the lower serum Hcy level is strongly associated with in-hospital unfavorable outcome. Further prospective studies of Hcy natural history and managements in bAVMs are required, which would be valuable for evaluating the disease-modifying efficacy of oral nutritional supplements in bAVM patients.

## 1. Introduction

Brain arteriovenous malformations (bAVMs) are well known as congenitally abnormal conglomerations of dilated feeding arteries and draining veins without intervening capillary beds [1, 2]. Although bAVM occurs in approximately 15/100,000 persons, it is still the leading cause of hemorrhagic stroke in young people and often results in a high incidence of neurological morbidity and mortality [2]. Prediction of outcomes is mostly based on demographic, clinical, and radiographic markers, while the information on routine laboratory examinations is inadvertently ignored.

Hyperhomocysteinemia (HHcy) is an elevation of serum homocysteine (Hcy) concentration due to the methionine metabolic disorder [3]. Characterized by the atherogenic and thrombogenic effects, Hcy is recognized as the independent risk factor for the major adverse cardiovascular and cerebrovascular events (MACCEs) and mortality [4, 5]. Apparently, previous studies have focused on hemorrhagic stroke and unfavorable outcomes [5, 6]. Moreover, some studies suggested that Hcy disorders and hemorrhagic bAVMs shared similar underlying mechanisms in terms of hemodynamics [7–9] and molecular abnormalities [10–14]. However, the above-mentioned studies were based on other hemorrhagic subtypes and animal experiments; the clinical significance of Hcy in patients with bAVM has not been discussed yet.

The present study was investigated to take an exploratory look into the association between serum Hcy levels in patients with bAVM and their impact on neurological outcome during hospitalization.

## 2. Materials and Methods

### 2.1. Study Design and Participants

We retrospectively reviewed patients diagnosed with bAVMs at the Department of Neurosurgery, Beijing Tiantan Hospital, from January 2019 to August 2020. The study was approved by the Institutional Review Board of our institution, and informed consent was waived considering the retrospective design of the study.

**Figure 1:**
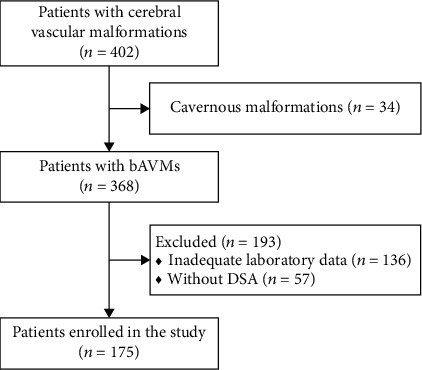
Flow diagram of the study participants. bAVMs: brain arteriovenous malformations; DSA: digital subtraction angiography.

Among 402 patients with cerebral vascular malformations admitted to our hospital between January 2019 and August 2020, 332 patients were diagnosed with bAVMs using digital subtraction angiography (DSA) or magnetic resonance imaging (MRI). Patients with inadequate laboratory or DSA data were excluded. Finally, a total of 175 patients were included in the study (Figure 1). The enrolled patients were divided into two groups according to the neurological outcome at discharge (Group 1, modified Rankin Scale [mRS] ≤ 2; Group 2, mRS > 2).

### 2.2. Data Collection and Outcome Evaluation

Demographic data, medical and personal history, prior treatments, primary symptoms, bAVM radiographic characteristics, clinical features, laboratory results, and treatment modality were obtained. Medical and personal history including hypertension, diabetes mellitus, hyperlipidemia, cigarette smoking, and alcohol drinking were recorded. The treatment history included embolization, radiosurgery, and microsurgery. Primary symptoms were summarized as hemorrhage, seizure, and neurological dysfunction. Radiographic characteristics included volume of lesion, deep and eloquent location, venous drainage patterns, and associated aneurysms. The AVM volume was calculated by (*a* × *b* × *c*)/2. Spetzler-Martin (SM) grading scale was evaluated to stratify the bAVMs. The treatment modalities were dichotomized based on the involvement of microsurgery. The clinical status was determined by the mRS score at admission and discharge. Neurological disability (mRS > 2) was defined as the clinical outcome. The neurological assessment at discharge was considered a short-term outcome.

Heart rate and blood pressure were also obtained. Body mass index (BMI) was calculated as weight (kg)/[height (m)]^2^. Fast venous blood samples were collected in the morning after admission for all bAVM patients. Levels of blood glucose, albumin (ALB), creatinine, uric acid (UA), total cholesterol (TC), triglyceride (TG), high-density lipoprotein cholesterol (HDL-C), low-density lipoprotein cholesterol (LDL-C), apolipoprotein A (ApoA), apolipoprotein B (ApoB), and Hcy were measured using enzymatic methods. Serum Hcy ≥ 15.0 *μ*mol/L was defined as HHcy [15].

### 2.3. Statistical Analysis

Categorical variables were expressed as frequencies, and continuous variables were presented with a mean (standard deviation (SD)) or median (interquartile range (IQR)). A chi-square test or Fisher's exact test was performed to compare categorical variables between groups. Continuous variables were compared by two-tailed Student's *t*-test or Mann–Whitney *U* test. The association between variables and Hcy quartiles was analyzed using the Cochran-Armitage test for bivariate variables and Spearman's rank correlation test for continuous variables. Logistic regression analyses were conducted to identify the potential risk factors for short-term neurological outcome. Variables achieving *P* < 0.10 in univariate analysis were included in the multivariate analysis. *P* value < 0.05 was considered statistical significance. Statistical analyses were performed using SPSS 26.0 (IBM, New York, USA).

## 3. Results

Four hundred and two cerebral vascular malformation patients were identified. After excluding 34 cavernous malformations and 193 patients with incomplete data, a total of 175 bAVM patients were enrolled in our study (Figure 1).

### 3.1. Baseline Characteristics of bAVM Patients

The baseline characteristics of bAVM patients are shown in Table 1. The mean age at diagnosis was 29.6 years, with a male-to-female ratio of 1.3 : 1. Thirty-one cases (17.7%) were at cigarette smoking status, and 17 cases (9.7%) were current alcohol abused. Sixty cases (34.3%) had received prior treatments, including embolization in 43 (24.6%), radiosurgery in 12 (6.9%), and microsurgery in 5 (2.9%). Hemorrhage (56.6%) occurred as the most common primary symptom, followed by seizure (20.0%) and neurological dysfunction (17.7%). Poor neurological status (mRS > 2) was observed in 15.4% of cases (*n* = 27) on admission. Twenty-eight cases (16.0%) harbored SM grade IV-V lesions. The average volume of lesions was 8.6 cm^3^. Deep locations were found in 51 cases (29.1%), and eloquent areas were involved in 52.0% of cases (*n* = 91). 34.3% of cases (*n* = 60) had deep venous drainage, and 12.6% of cases (*n* = 22) had associated aneurysms. HHcy was identified in 32.6% of cases (*n* = 57). According to the treatment modalities, microsurgery was involved in 78 cases (44.6%). Compared with Group 1, patients in Group 2 more likely presented with hemorrhage (*P* = 0.001), and exhibited poor neurological status (mRS > 2) (*P* < 0.001). In terms of the radiographic characteristics, the proportion of low SM grade (I-II) was found a significant difference between groups (*P* = 0.012). Patients with unfavorable outcomes were more located in deep areas (*P* = 0.023) and drained by deep veins (*P* = 0.026). In addition, higher level of glucose (*P* = 0.034), lower level of albumin (*P* = 0.019), and Hcy (*P* = 0.023) were found in Group 2.

### 3.2. Characteristics of bAVM Patients according to Hcy Quartiles

Clinical variables according to Hcy quartiles are summarized in Table 2. A linear association was observed between Hcy level and male sex (*P* < 0.001). Serum Hcy level was also related to age (*P* = 0.011), cigarette smoking (*P* = 0.001), and seizure manifestation (*P* = 0.034). Furthermore, Hcy was correlated with short-term neurological outcome (*P* = 0.027). Although the hemorrhagic risk was not a significant difference between Hcy quartiles (*P* = 0.052), a relatively lower incidence of rupture occurred in the groups of higher Hcy level (Q1: 61.4%; Q2, 59.1%; Q3, 58.1%; and Q4, 47.7%). Other variables were similar across groups (*P* > 0.05 for all).

### 3.3. Analysis of Neurological Outcomes

**Figure 2:**
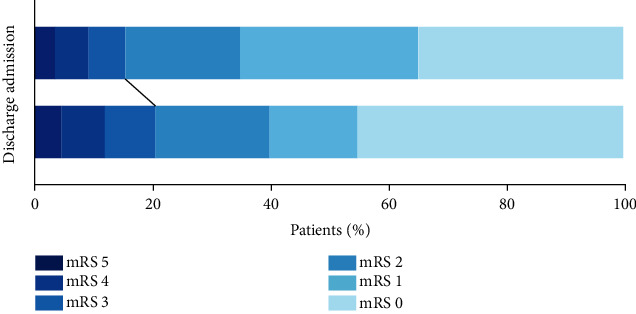
Comparison of mRS score in bAVM patients at admission and discharge. Thirty-six patients (20.6%) were disabled (mRS > 2) at discharge, with no significant difference compared with admission (*P* = 0.211). mRS: modified Rankin Scale.

Neurological outcomes of bAVM patients were analyzed. Thirty-six patients (20.6%) were disabled (mRS > 2) at discharge, with no significant difference compared with admission (*P* = 0.211) (Figure 2). No in-hospital mortality occurred during hospitalization. According to the variation of mRS scores, most patients experienced an improved or unchanged neurological status in the short-term (81.7%, *n* = 143).

Risk factors for the neurological outcome of bAVM patients were identified (Table 3). Univariate analysis showed that hemorrhagic presentation, admission mRS score, AVM volume, deep location, deep venous drainage, associated aneurysms, glucose, albumin, TC, and Hcy were related to the neurological outcome. After additionally adjusting for age and male sex, hemorrhagic presentation (OR [odds ratios] 5.360, 95% CI [confidence intervals] 1.208-23.790; *P* = 0.027), admission mRS score (OR 2.225, 95% CI 1.402-3.530; *P* = 0.001), AVM volume (OR 1.024, 95% CI 1.004-1.046; *P* = 0.021), deep venous drainage (OR 8.813, 95% CI 1.965-39.534; *P* = 0.004), associated aneurysms (OR 5.711, 95% CI 1.389-23.488; *P* = 0.016), glucose (OR 1.897, 95% CI 1.115-3.229; *P* = 0.018), and Hcy (OR 0.838, 95% CI 0.720-0.976; *P* = 0.023) were shown to be significantly associated with short-term disability in the multivariate analysis.

## 4. Discussion

Our retrospective study demonstrated the potential for serum levels of Hcy to serve as an objective biomarker for prognosticating short-term neurological outcomes in patients with bAVMs. The lower serum Hcy level predicted a higher risk of an unfavorable outcome. Furthermore, we identified a positive correlation between HHcy and symptomatic seizures on admission.

Admittedly, previously verified factors, including hemorrhagic presentation, admission mRS score, AVM volume, deep venous drainage, and associated aneurysms, have been broadly studied [1, 16]. Sometimes, the evaluation between such indicators and prognosis will be subjective. When the classic Spetzler-Martin (SM) grading system [17], supplementary scale [18], and even with evolving fMRI-based HDVL grading scale combined with lesion-to eloquence distance (LED) are added [19], the evaluation becomes accurate but tedious at the same time. And there are some objectively advanced biomarkers, such as S100B, matrix metalloproteinase-9 (MMP-9), interleukin-1 beta (IL-1*β*), vascular endothelial growth factor (VEGF), and N6-methyladenosine methyltransferase 3 (METTL3) [12, 20–22], which were proposed to be the predictors for the in-hospital complications and neurological outcomes. However, these indicators were unable to directly provide guidance for clinical practice. The underlying diagnostic and predictable values of laboratory biomarkers routinely examined after admission are required to be further excavated. To our best knowledge, we first associated the serum Hcy level with the short-term neurological outcome. After being adjusted for other known factors, the predicted value would still be significant (OR 0.838, 95% CI 0.720-0.976; *P* = 0.023).

For decades, the abnormal Hcy level was acknowledged as the independent risk factors for the onset of ischemic stroke in several large scale multicenter trials [3, 5, 23, 24]. Afterward, the relationship between hemorrhagic stroke and HHcy has been subsequently studied but reached contrary conclusions. Li et.al found that elevated Hcy was correlated with hemorrhagic stroke by1.94-fold compared to the controls [5]. Larger hematoma volume in patients with thalamoganglionic intracerebral hemorrhage (ICH) associated with the elevated Hcy level was identified by Hu et al. [25]. Furthermore, Zhang and his colleagues developed a model consisted of Hcy level to identify high-risk groups for predicting recurrent ICH, which can facilitate the preemptive clinical intervention [26]. Zhou et al. conducted a meta-analysis including 667 ICH patients and demonstrated that the Hcy level is positively associated with a high risk of ICH [6]. Nevertheless, two cohorts in China found that HHcy correlated with a lower risk of hemorrhagic stroke [27]. For other hemorrhagic subtypes, such as subarachnoid hemorrhage (SAH), researchers gained different findings. In 2001, McEvoy et al. revealed no association between the Hcy level and the etiology of SAH [28]. In favor of the upper conclusion, Grobelny et al. found no correlations between Hcy and delayed cerebral ischemia (DCI), while the gain-of-function polymorphisms of the cystathionine *β*-synthase (CBS) gene could reduce the risk of DCI after aneurysmal SAH and improve the outcome at discharge [29]. Moreover, the CARAS study showed that increased CBS activity may exert its neuroprotective effects in altering the Hcy level and then improve clinical outcomes [30]. Another gene polymorphism methylenetetrahydrofolate reductase (MTHFR) C677T might contribute to a higher Hcy level to impair the clinical outcomes in patients with SAH [31]. Unlike their predecessors, Dhandapani et al. explored a reverse epidemiology paradox, where the higher Hcy level appears to be a significant association with both survival and favorable neurological outcomes [32].

In the present study, we reached a similar conclusion as SAH groups. Higher serum Hcy levels seemed to be a survival advantage in patients with bAVMs. We supposed that the lower serum Hcy level attributed to the activation in the cascade usage of Hcy's endogenously thrombotic effect after the hemorrhagic bAVM. As an intermediate metabolic product in the circle involving folate and vitamins, Hcy could be modifiable [33]. Two randomized controlled trials showed that folic acid/vitamin B12 or vitamin B6 had no effect of treatment on total mortality [3, 4]. However, Wang et al. performed a meta-analysis showing effective reduction in patients with stroke in primary prevention [34]. The conflicts between these studies were an account of the baseline folate consumption. The management of oral nutritional supplements on Hcy in the bAVMs group needs further investigation.

Another independent prominent predictor for the unfavorable neurological outcome is the higher serum glucose level (OR 1.897, 95% CI 1.115-3.229; *P* = 0.018). The same results were reached in the hemorrhagic subtypes, including ICH and SAH [35, 36].

Some clinical features of bAVMs determined by this study differed from those in previous reports. We demonstrated a statistical significance between Hcy quartiles and seizure manifestation (*P* = 0.034), which can induce unfavorable outcomes at discharge in the patients with bAVMs. The relationship between Hcy and seizure was first proposed in an intrathecal chemotherapy-treated boy who suffered from leukemia [37]. And any transient elevation of Hcy may be related to the seizure risk [38, 39]. Although it is hard to determine whether the elevated Hcy is the cause or the result of a seizure, we offered neurosurgeons a new insight into the prophylactic and off-label utility of antiepileptic drugs.

There are some limitations that should be acknowledged. First, due to the nature of the respective study, we are aware of the fact that its design is one of the considerations which should be improved in the future. This study's small sample size is the second considerable limitation because larger samples are preferred for relevant studies. As such, findings should be interpreted with caution until further high-level prospective studies or larger data sets are available. Third, the baseline dietary and folate status of the patients were undocumented. Though the population was under established policies of population folate supplementation for the few decades nationwide, we enrolled patients before the policy was carried out, which could be a confounding variable. Fourth, the outcomes were only measured at the discharge point and were not conducted at the 3-month follow-up. This is our ongoing study of enrolled patients, and we expect to make some progress in the recent future. Further, longer follow-up studies should be conducted to demonstrate the clinical relevance between the serum Hcy level and long-term outcomes.

## 5. Conclusions

In conclusion, our results indicated that the lower serum Hcy level is strongly associated with in-hospital unfavorable outcomes. Further prospective studies of Hcy natural history and management in the bAVMs groups are needed, which would be valuable for evaluating the disease-modifying efficacy of oral nutritional supplements in bAVM patients.

## Figures and Tables

**Table 1:** Baseline characteristics of bAVM patients between groups.

Variables	Total (*n* = 175)	Group 1^#^ (*n* = 139)	Group 2^#^ (*n* = 36)	*P* value
Age (y), mean (SD)	29.6 (14.7)	29.9 (14.6)	28.6 (15.1)	0.635
Sex, male (%)	100 (57.1)	78 (56.1)	22 (61.1)	0.589
Medical and personal history (%)				
Hypertension	14 (8.0)	9 (6.5)	5 (13.9)	0.264
Diabetes mellitus	5 (2.9)	3 (2.2)	2 (5.6)	0.597
Hyperlipidemia	1 (0.6)	1 (0.7)	0 (0)	>0.999
Cigarette smoking	31 (17.7)	25 (18.0)	6 (16.7)	0.853
Alcohol drinking	17 (9.7)	13 (9.4)	4 (11.1)	0.999
Prior treatments (%)				
Embolization	43 (24.6)	32 (23.0)	11 (30.6)	0.349
Radiosurgery	12 (6.9)	8 (5.8)	4 (11.1)	0.445
Microsurgery	5 (2.9)	5 (3.6)	0 (0)	0.585
Primary symptom (%)				
Hemorrhage	99 (56.6)	70 (50.4)	29 (80.6)	0.001^∗^
Seizure	35 (20.0)	30 (21.6)	5 (13.9)	0.304
Neurological dysfunction	31 (17.7)	24 (17.3)	7 (19.4)	0.760
Admission mRS > 2 (%)	27 (15.4)	6 (4.3)	21 (58.3)	<0.001^∗^
Radiographic characteristics				
Spetzler-Martin grade (%)				
I-II	91 (52.0)	79 (56.8)	12 (33.3)	0.012^∗^
III	56 (32.0)	41 (29.5)	15 (41.7)	0.163
IV-V	28 (16.0)	19 (13.7)	9 (25.0)	0.098
Volume (cm^3^), median (IQR)	8.6 (4.1-25.6)	8.5 (4.1-25.6)	8.7 (4.3-26.5)	0.388
Deep location (%)	51 (29.1)	35 (25.2)	16 (44.4)	0.023^∗^
Eloquent location (%)	91 (52.0)	68 (48.9)	23 (63.9)	0.109
Deep venous drainage (%)	60 (34.3)	42 (30.2)	18 (50.0)	0.026^∗^
Associated aneurysms (%)	22 (12.6)	14 (10.1)	8 (22.2)	0.093
Clinical features, mean (SD)				
Heart rate, bpm	80 (10)	80.3 (9.8)	77.0 (9.3)	0.312
SBP (mm Hg)	120 (19)	119.5 (17.1)	121.4 (25.7)	0.666
DBP (mm Hg)	77 (11)	76.8 (10.4)	80.1 (12.8)	0.162
BMI (kg/m^2^)	22.5 (4.5)	22.6 (4.5)	22.2 (4.5)	0.654
Laboratory results, median (IQR)				
Glucose (mmol/L)	4.7 (4.4-5.1)	4.7 (4.4-5.1)	4.9 (4.3-5.2)	0.034^∗^
Albumin (g/L)	43.9 (41.4-46.1)	44.3 (41.4-46.1)	42.5 (41.1-46.2)	0.019^∗^
Creatinine (*μ*mol/L)	58.5 (46.5-68.3)	58.6 (46.5-68.3)	55.7 (46.8-68.2)	0.591
UA (*μ*mol/L)	309.7 (254.9-363.6)	314.0 (254.9-362.2)	302.2 (247.9-362.9)	0.269
TC (mmol/L)	4.2 (3.6-4.8)	4.3 (3.6-4.8)	4.1 (3.6-4.9)	0.072
TG (mmol/L)	1.0 (0.7-1.5)	1.0 (0.7-1.5)	1.0 (0.7-1.5)	0.622
HDL-C (mmol/L)	1.2 (1.0-1.5)	1.2 (1.0-1.5)	1.2 (1.0-1.5)	0.480
LDL-C (mmol/L)	2.6 (2.0-3.1)	2.6 (2.0-3.1)	2.4 (1.9-3.1)	0.191
ApoA (g/L)	1.3 (1.1-1.4)	1.3 (1.1-1.4)	1.2 (1.1-1.4)	0.690
ApoB (g/L)	0.8 (0.7-1.0)	0.8 (0.7-1.0)	0.8 (0.7-1.0)	0.203
Hcy (*μ*mol/L)	12.5 (9.9-16.6)	13.0 (9.9-16.6)	10.2 (9.8-15.9)	0.023^∗^
HHcy (%)	57 (32.6)	49 (35.3)	8 (22.2)	0.137
Treatment modality				0.137
Microsurgery involvement	78 (44.6)	58 (41.7)	20 (55.6)	
Nonmicrosurgery involvement	97 (55.4)	81 (58.3)	16 (44.4)	

bAVM: brain arteriovenous malformation; SD: standard deviation; mRS: modified Rankin Scale; IQR: interquartile range; SBP: systolic blood pressure; DBP: diastolic blood pressure; BMI: body mass index; UA: uric acid; TC: total cholesterol; TG: triglyceride; HDL-C: high-density lipoprotein cholesterol; LDL-C: low-density lipoprotein cholesterol; ApoA: apolipoprotein A; ApoB: apolipoprotein B; Hcy: homocysteine; HHcy: hyperhomocysteinemia. ^#^Group 1, modified Rankin Scale [mRS] ≤ 2. Group 2, mRS > 2. ^∗^*P* < 0.05, significant difference.

**Table 2:** Characteristics of bAVM patients according to Hcy quartiles.

Variables	Hcy quartiles^a^ (*μ*mol/L)					*P* trend
	All (*n* = 175)	Q1 (*n* = 44)	Q2 (*n* = 44)	Q3 (*n* = 43)	Q4 (*n* = 44)	
Age (y), mean (SD)	29.6 (14.7)	24.0 (15.7)	30.8 (14.2)	34.4 (13.4)	29.3 (13.8)	0.011^∗^
Sex, male (%)	100 (57.1)	13 (29.5)	22 (50.0)	29 (67.4)	36 (81.8)	<0.001^∗^
Medical history (%)						
Hypertension	14 (8.0)	2 (4.5)	2 (4.5)	6 (14.0)	4 (9.1)	0.210
Diabetes mellitus	5 (2.9)	1 (2.3)	1 (2.3)	1 (2.3)	2 (4.5)	0.540
Hyperlipidemia	1 (0.6)	0 (0)	0 (0)	1 (2.3)	0 (0)	0.653
Cigarette smoking	31 (17.7)	1 (2.3)	7 (15.9)	10 (23.3)	13 (29.5)	0.001^∗^
Alcohol drinking	17 (9.7)	0 (0)	4 (9.1)	9 (20.9)	4 (9.1)	0.052
Primary symptom (%)						
Hemorrhage	99 (56.6)	27 (61.4)	26 (59.1)	25 (58.1)	21 (47.7)	0.210
Seizure	35 (20.0)	5 (11.4)	8 (18.2)	9 (20.9)	13 (29.5)	0.034^∗^
Neurological dysfunction	31 (17.7)	9 (20.5)	8 (18.2)	8 (18.6)	6 (13.6)	0.416
Admission mRS > 2 (%)	27 (15.4)	13 (29.5)	3 (6.8)	6 (14.0)	5 (11.4)	0.052
AVM characteristics						
Spetzler-Martin grade (%)						0.885
I-II	91 (52.0)	19 (43.2)	27 (61.4)	23 (53.5)	22 (50.0)	
III	56 (32.0)	17 (38.6)	12 (27.3)	13 (30.2)	14 (31.8)	
IV-V	28 (16.0)	8 (18.2)	5 (11.4)	7 (16.3)	8 (18.2)	
Volume (cm^3^), median (IQR)	8.6 (4.1-25.6)	11.4 (5.0-29.7)	7.3 (4.5-18.7)	8.6 (3.1-22.7)	11.5 (4.1-31.8)	0.596
Deep location (%)	51 (29.1)	13 (29.5)	11 (25.0)	12 (27.9)	15 (34.1)	0.338
Eloquent location (%)	91 (52.0)	26 (59.1)	21 (47.7)	23 (53.5)	21 (47.7)	0.400
Deep venous drainage (%)	60 (34.3)	15 (34.1)	13 (29.5)	16 (37.2)	16 (36.4)	0.687
Associated aneurysms (%)	22 (12.6)	6 (13.6)	5 (11.4)	6 (14.0)	5 (11.4)	0.849
Microsurgery involvement (%)	78 (44.5)	23 (52.3)	19 (43.2)	18 (41.9)	18 (40.9)	0.145
Postoperative stroke (%)	13 (7.4)	5 (11.4)	1 (2.3)	6 (14.0)	1 (2.3)	0.373
Short-term neurological disability (%)	36 (20.6)	17 (38.6)	4 (9.1)	9 (20.9)	6 (13.6)	0.027^∗^

bAVM: brain arteriovenous malformation; Hcy: homocysteine; SD: standard deviation; mRS: modified Rankin Scale; IQR: interquartile range. ^∗^*P* < 0.05, significant difference. ^a^Serum levels of Hcy in quartiles: Q1, <9.9 *μ*mol/L; Q2, 9.9-12.7 *μ*mol/L; Q3, 12.8-16.6 *μ*mol/L; and Q4, ≥16.1 *μ*mol/L.

**Table 3:** Logistic regression analysis on the neurological outcome.

Variables	Univariate analysis			Multivariate analysis		
	OR	95% CI	*P* value	OR	95% CI	*P* value
Age	0.994	0.969-1.019	0.633	1.011	0.969-1.055	0.609
Male sex	1.229	0.581-2.599	0.590	2.528	0.659-9.698	0.177
Medical history						
Hypertension	2.330	0.729-7.441	0.153			
Diabetes mellitus	2.667	0.429-16.594	0.293			
Cigarette smoking	0.912	0.343-2.424	0.853			
Alcohol drinking	1.212	0.370-3.966	0.751			
Primary symptoms						
Hemorrhage	4.084	1.677-9.943	0.002	5.360	1.208-23.790	0.027^∗^
Seizure	0.586	0.210-1.637	0.308			
Neurological dysfunction	1.157	0.454-2.947	0.760			
Admission mRS score	2.496	1.803-3.457	<0.001	2.225	1.402-3.530	0.001^∗^
AVM characteristics						
Volume	1.018	1.005-1.032	0.007	1.024	1.004-1.046	0.021^∗^
Deep location	2.377	1.111-5.087	0.026	0.927	0.238-3.613	0.913
Eloquent location	1.847	0.866-3.938	0.112			
Deep venous drainage	2.310	1.094-4.874	0.028	8.813	1.965-39.534	0.004^∗^
Associated aneurysms	2.551	0.976-6.666	0.056	5.711	1.389-23.488	0.016^∗^
Clinical features						
Heart rate	0.980	0.944-1.019	0.311			
SBP	1.004	0.985-1.025	0.664			
DBP	1.024	0.990-1.059	0.164			
BMI	0.980	0.899-1.069	0.652			
Laboratory results						
Glucose	1.579	1.151-2.166	0.005	1.897	1.115-3.229	0.018^∗^
Albumin	0.900	0.816-0.993	0.035	0.968	0.820-1.142	0.697
Creatinine	0.995	0.972-1.018	0.667			
UA	1.001	0.999-1.002	0.325			
TC	0.643	0.421-0.983	0.042	0.194	0.033-1.129	0.068
TG	0.944	0.636-1.403	0.776			
HDL-C	1.639	0.870-3.088	0.126			
LDL-C	0.692	0.441-1.086	0.109			
ApoA	0.868	0.228-3.303	0.836			
ApoB	0.253	0.040-1.595	0.143			
Hcy	0.937	0.878-1.001	0.055	0.838	0.720-0.976	0.023^∗^
Microsurgery involvement	1.746	0.834-3.654	0.139			

OR: odds ratio; CI: confidence intervals; AVM: arteriovenous malformation; SBP: systolic blood pressure; DBP: diastolic blood pressure; BMI: body mass index; UA: uric acid; TC: total cholesterol; TG: triglyceride; HDL-C: high-density lipoprotein cholesterol; LDL-C: low-density lipoprotein cholesterol; ApoA: apolipoprotein A; ApoB: apolipoprotein B; Hcy: homocysteine. ^∗^*P* < 0.05, significant difference.

## Data Availability

The data used to support the findings of this study are available from the corresponding author upon reasonable request.
